# A commentary on ‘Incidence and cost of vertebral fracture in urban China: a five-year population-based cohort study’

**DOI:** 10.1097/JS9.0000000000000583

**Published:** 2023-07-06

**Authors:** Lan Shen, Kun Luo, Xuelin Deng, Jingjing Liu

**Affiliations:** a Department of Emergency Medicine; b Department of Spinal Surgery, Southern Central Hospital of Yunnan Province (First People’s Hospital of Honghe State), Yunan, People’s Republic of China

*Dear Editor,*

Osteoporosis is the most common systemic and metabolic skeletal disease, characterized by low bone mass, deterioration of bone tissue, disruption of bone architecture, compromised bone strength, and increased risk of fracture. Osteoporotic vertebral fracture (OVF) is the most common and serious osteoporotic fracture and is a major health problem in many people^1^. The incidence of OVF will increase incrementally due to the aging society, which will increase the economic burden.

Zheng *et al*.^2^ conducted a population-based cohort study by using Urban Employee Basic Medical Insurance (UEBMI) and Urban Resident Basic Medical Insurance (URBMI) data in China from 2013 to 2017. They found that the incidence and cost of OVF were increasing year by year and suggested that more attention should be given to the management of osteoporosis to prevent osteoporotic fractures.

Underdiagnosis of OVF is a worldwide problem. In a multinational study assessing the accuracy of radiographic diagnosis of vertebral fractures, the false-negative rates were 34% globally (from 27 to 45%), despite good image quality, the same semiquantitative technique, and the quality assurance manual for fracture assessment^3^. Possible explanations include radiologists often focus on more acute, significant, and urgent findings related to the heart and lungs; radiologists lack sufficient knowledge of osteoporosis and sufficient training for fracture recognition; radiologists diagnose OVF according to the characteristic findings of traumatic fracture and do not recognize deformed vertebrae as manifestations of osteoporotic fracture.

How can we improve the radiological diagnosis of OVF? Because many OVFs are present on radiographs obtained for other reasons and are unsuspected clinically, radiologists must use a standardized approach to the diagnosis of OVF, such as vertebral fracture assessment. Genant’s semiquantitative assessment and quantitative assessments are two major approaches to the identification of vertebral fractures^4^. Both approaches have moderate sensitivity and specificity for identifying OVF and have high reproducibility. Purely quantitative morphometric assessment relies entirely upon placing six points in each vertebral body to calculate the anterior, middle, and posterior heights and their ratios. One area of concern with this approach is that thoracic vertebrae are usually wedge-shaped and might be falsely diagnosed as mild fractures by morphometric assessment, especially in the presence of kyphosis due to osteoarthritis, for example. Thus, quantitative morphometric assessment is not recommended in routine clinical settings. Genant’s semiquantitative assessment is a basis for a standardized interpretation of OVF severity and is simple for radiologists and orthopedic surgeons to learn and apply in clinical practice. Genant’s semiquantitative assessment of OVFs can be performed on lateral radiographs of the thoracolumbar spine or lateral spine images obtained with dual-energy X-ray absorptiometry (DXA) and has been recommended for identification of OVFs.

Women are at comparably high risk for OVF. Therefore, it seems to be reasonable to look for osteoporosis in female patients over 60 as an underlying disease after a sustained fracture. Patients suffering from a major osteoporotic fracture of the spine have a significantly increased relative risk of sustaining another fracture within a short period of time. Prevention of further new fractures is of paramount importance for patients with existing OVF.

## Ethical approval

Not applicable.

## Sources of funding

None.

## Author contribution

L.S.: writing; K.L.: study design; X.D.: data analysis; J.L.: data collection.

## Conflicts of interest disclosure

No conflicts of interest.

## Research registration unique identifying number

Not applicable.

## Guarantor

Jingjing Liu.
